# EXAMINING THE EXPERIENCES AND UNIQUE CARE NEEDS OF SPEC POPS IN AL: FACILITATORS AND BARRIERS TO MAINTAINING THE SELF

**DOI:** 10.1093/geroni/igae098.4177

**Published:** 2024-12-31

**Authors:** Jeffrey Lentz, Alexis Bender, Candace Kemp, Molly Perkins

**Affiliations:** Emory University, Atlanta, Georgia, United States; Emory University, Atlanta, Georgia, United States; Georgia State University, Atlanta, Georgia, United States; Emory University, Atlanta, Georgia, United States

## Abstract

The assisted living (AL) population is growing more diverse. This diversity is reflected in the increasing use of AL by African Americans, Latinos, and other cultural groups who have traditionally “cared for their own,” as well as a rising number of members of other special populations (e.g., sexual minorities and persons aging with HIV) requiring and using residential care. As part of a 5-year study of care at the end of life in AL funded by the National Institute on Aging (R01AG047408), we use the Sort and Sift, Think and Shift method to investigate the experiences and unique care needs of AL residents who represent various special populations within our sample. Data include in-depth and informal interviews with residents. These cases (three females and two males) reflect intersecting social statuses (e.g., race, class, ethnicity, sexual orientation, illness, and disability). Our analysis identified facilitators and barriers to maintaining the self. These statuses make them particularly vulnerable to stigma and other challenges that adversely affect their quality of life, including trauma earlier in life, language barriers, cultural marginalization, discrimination, and inadequate social support. Strengths that contribute to their ability to negotiate these risks and maintain a positive sense of self include religiosity and spirituality, as well as having a sense of community or group pride (e.g., as an HIV survivor or member of a racial/ethnic group). Findings have implications for interventions to improve the quality of life at the end of life for residents who represent members of special, often hidden populations.

